# Influence of Coping and Self-Efficacy in Inflammatory Bowel Disease

**DOI:** 10.3390/healthcare11081113

**Published:** 2023-04-13

**Authors:** Estela Muñoz González, Carlos Durantez-Fernández, Lucía Pérez-Pérez, María José de Dios-Duarte

**Affiliations:** 1 Madrilenian Health Service (SERMAS), 28046 Madrid, Spain; 2 Nursing Department, Faculty of Nursing, University of Valladolid, 47005 Valladolid, Spain; 3 Castilla y León Health Service (SACYL), 47007 Valladolid, Spain; 4 Nursing Care Research (GICE), University of Valladolid, 47005 Valladolid, Spain

**Keywords:** coping, self-efficacy, inflammatory bowel disease

## Abstract

(1) Background: Coping includes the specific cognitive processes and behaviours that the patient uses when faced with the stress of living with a chronic disease. Self-efficacy is the knowledge that individuals have about their abilities and their confidence to face a problem or cope with a situation (disease). The aim of this study was to explore the role of coping and self-efficacy in inflammatory bowel disease. (2) Materials and Methods: A total of 92 participants were included (33 had been diagnosed with Crohn’s disease, 23 with ulcerative colitis and 36 were healthy participants). The Coping Strategies Inventory was used to measure which coping strategies were employed, differentiating them as active or passive. The General Self-Efficacy Scale was used to measure self-efficacy. (3) Results: The results indicate that people with inflammatory bowel disease used strategies related to passive coping more than healthy people (mean of 36.39 ± 13.92 vs. 29.77 ± 10.70, *p* = 0.017). Additionally, people with inflammatory bowel disease used social withdrawal more than healthy participants (mean of 8.30 ± 5.07 vs. 4.47 ± 4.17, *p* < 0.001). In addition, there are significant differences in emotion-focused engagement coping strategies. People with inflammatory bowel disease used this strategy less than healthy people (mean of 21.77 ± 7.75 vs. 25.03 ± 7.00, *p* = 0.044). Finally, healthy participants used the emotion-focused disengagement strategy less than those diagnosed with inflammatory bowel disease (mean of 9.81 ± 7.74 vs. 15.61 ± 10.14, *p* = 0.004). (4) Conclusions: Actions aimed at the development of active coping strategies and patient socialisation must be included in the treatment of inflammatory bowel disease.

## 1. Introduction

Inflammatory bowel disease (IBD) basically involves two chronic immune disorders of the digestive tract characterised by acute and chronic inflammation of the same. These two conditions are ulcerative colitis and Crohn’s disease. A third type of inflammatory bowel disease has also been established and is referred to as “unclassified inflammatory bowel disease”, or “indeterminate colitis”. This study, however, only focuses on Crohn’s disease and ulcerative colitis, since the collection of data for indeterminate colitis is complicated both by its definitive diagnosis and the percentage of cases that exist. Both ulcerative colitis and Crohn’s disease have gained special importance in recent years due to the increase in the number of cases and the great economic and social impact they generate. Thus, it is essential to study these diseases in greater depth in order to give professionals further knowledge to help them provide better care and attention to these patients [1,2,3].

These diseases significantly affect the lives of diagnosed patients, due to their chronic and sometimes disabling nature with the occurrence of outbreaks and remissions of the pathology [4]. Furthermore, there are added complications such as high surgical morbidity, the risk of developing colorectal cancer—mainly in the case of Crohn’s disease—and the limited response to treatments [5,6].

Psychosocial factors affect the course and evolution of these diseases in such a way that, by identifying this influence, actions can be taken to improve the situation of diagnosed patients [3,7]. Interestingly, during the course of chronic disease, it has been proven that knowledge of people’s coping strategies plays a fundamental role in adapting to the same.

Coping strategies are the specific cognitive processes and behaviours that the patient uses when faced with the stress of living with a chronic disease [8,9].

In the case of chronic pathologies such as inflammatory bowel disease, assessment of the patient’s stressful situation will establish the way it can or cannot be dealt with and adapted to [10]. Thus, the different coping strategies used by patients help to explain the differences during the course of the disease, their ability to adapt to it and survival [11].

When individuals see the situation as a challenge, they will face it and respond to it. However, when individuals see the situation as a threat or possibly harmful to them, they will doubt their ability to cope with it and fear being harmed. These are inadequate responses to stress [12]. The use of active coping therefore requires greater effort from patients, since they must take action, while passive coping implies an inactive attitude.

Generically, coping refers to the behavioural and psychological efforts made by individuals to tolerate, manage or minimise adverse stressful situations [13]. According to Lazarus and Folkman [14], there are two types of coping strategies: those focused on problem solving, and those focused on emotions.

Problem-focused strategies are normally used in conditions of controllable stress, while those focused on emotions are usually used in conditions that the patient considers to be uncontrollable, such as the risk of death, the development of cancer and so forth [15]. Thus, task-oriented or action-oriented strategies are focused on problem solving. Emotion-focused strategies, however, are those where efforts are directed at the emotional consequences of stressful situations rather than toward changing the situation, since the patient believes such change to be impossible [16].

The study conducted by Pourang and Besharat in 2011 showed that patients who had suffered myocardial infarction and used problem-focused coping strategies had a more effective recovery than those who used emotion-focused strategies [17].

In general, people who use active coping strategies when faced with a stressful situation are more inclined toward resolving the problem. They are more flexible and adapt better to situations than those who use passive coping strategies [18]. When active coping strategies are effective, they contribute to the quality of life, as well as the physical, psychological and social well-being of the individual, subsequently improving their adaptation [19,20].

On the other hand, patients who use passive coping strategies when faced with changes are usually invaded with fear, anxiety and discomfort since they feel threatened [21].

Regarding emotion-focused coping, a systematic review conducted in 2021 on irritable bowel syndrome and coping strategies [22] reported that this type of coping is associated with worse psychological outcomes, while the effect of problem-focused coping was, in some cases, associated with better psychological outcomes. Thus, maladaptive coping strategies were also associated to poor quality of life and psychiatric comorbidities.

Knowledge of coping strategies allows actions to be taken that transform non-adaptive coping mechanisms into adaptive ones, facilitating the patient’s adaptation to the disease [12]. The coping process can have positive consequences if stressful situations are overcome properly. This requires patients to increase their ability to adapt to the disease and see themselves as self-efficacious, thereby experiencing fewer negative psychological symptoms [16,23].

Self-efficacy or perceived efficacy is how the subject feels and personally judges his or her own abilities to handle life situations based on the resources he or she possesses, stimulating or inhibiting his or her conduct [24,25,26,27].

People with high levels of self-efficacy adapt better psychologically, socially and biologically to different life situations [28]. Perceived self-efficacy is therefore essential in coping with events that the person considers threatening so that the higher the self-efficacy, the lower the stress level and the greater the ability to adapt [29,30]. Having a high level of self-efficacy improves both the confidence that people have in themselves, as well as in their abilities [31,32], which also contributes to an increase in emotional well-being, quality of life and coping with stressful situations [33,34].

It has been shown that the perception of self-efficacy plays a fundamental role in adapting to the disease in patients with chronic diseases [33,35].

In addition, the perception of self-efficacy can influence the type of coping strategy used by the patient [34,36]. For example, patients with a high level of self-efficacy experiencing chronic pain rely more on the use of active rather than passive coping strategies. However, if the pathology generates a greater feeling of loss of control, then patients use passive strategies more frequently [37].

Case–control studies were carried out in our previous investigations that compared samples of people with Crohn’s disease in flare-up phase, latency phase and healthy people. As a result, we saw that people in flare-up phase were highly stressed and lacked control, making it impossible to carry out stress-reducing actions under those circumstances. In addition, they were the ones with the highest level of stress compared to patients in latency phase and healthy people. They also had the highest external locus of control levels [3,7,38]. On the other hand, statistically significant results were found in latency-phase patients as regards the external locus of control and stress. It was also observed that during the latency phase, low levels of stress and external locus of control generated a lower probability of an outbreak [3,39].

The locus of control is considered a personality trait that can be defined as the degree to which a subject considers that he or she has control over his or her life and, more specifically, over the various situations that arise. Thus, there are two types of locus of control. On the one hand, the internal locus of control, which is the subject’s belief that the events that occur to him or her are the result of his or her own actions, and on the other, the external locus of control, which is the subject’s belief that events that happen to him or her are the result of chance, fate, luck or the power and decisions of others [40].

Thus, the former believe that they can exercise control over their own lives, but the latter do not.

At this point, it is important to highlight that we are dealing with a belief, in the sense that the subject considers that the events occurring are the product of his or her own actions, yet whether the subject feels capable or not of carrying out the actions necessary to alter these events is another matter. This is what determines self-efficacy.

With respect to the conclusions of our previous research, as well as taking into account the results found regarding the locus of control and stress, it was confirmed that in latency-phase patients with a low external locus of control, both stress and the locus of control influenced the development of outbreaks so that the higher the levels of these two variables, the greater the probability of an outbreak occurring [3,39].

The research presented here is the next step in the study of the previously identified variables and their importance in the treatment of patients. In this paper, we delve into the psychological and social factors that prove to be very important in the treatment of inflammatory bowel disease, and expand our knowledge about them.

Based on the hypothesis that people with inflammatory bowel disease perceive less self-efficacy than healthy people and that they make more use of passive coping strategies, the main objective of our study was to explore the role of coping strategies and perceived self-efficacy in people with inflammatory bowel disease compared to healthy participants.

## 2. Materials and Methods

### 2.1. Participants

A case–control study was carried out using a sample consisting of a first group of patients diagnosed with inflammatory bowel disease in latency phase (not experiencing a flare-up), and a second group of healthy individuals without any noteworthy illnesses.

### 2.2. Procedure

The instructions recommended by the authors of the scales were given to all participants to ensure that questionnaires were correctly filled-in, especially those regarding coping and self-efficacy.

The sampling of patients with inflammatory bowel disease was carried out among the members of the Crohn’s and Ulcerative Colitis Associations’ Confederation (AC-CU Confederation, Spain), specifically from the associations belonging to the provinces of Madrid, Salamanca, the Canary Islands and Murcia (Spain). The research was presented to the patients, and the criteria required to be part of the study were explained. Questionnaires were then delivered to those patients who expressed their agreement to participate once it was clear that they had understood everything correctly. The patients were asymptomatic and were not being treated with prednisone. These patients said that they felt well and were not experiencing any abdominal pain. They passed 0–2 formed stools a day, without any rectal bleeding.

Healthy participants came from different environments and were either university students or people with basic, secondary, university or postgraduate studies. They were recruited from the same provinces as the participants in the diagnosed patients group. The procedure followed was the same as that for people suffering from inflammatory bowel disease. In all cases, they were asked to participate voluntarily and anonymously and provide their informed consent.

### 2.3. Inclusion and Exclusion Criteria

The inclusion criteria for participants diagnosed with IBD were the following: patients diagnosed with inflammatory bowel disease by a digestive system specialist; no other physiological disorders (coronary disorders, ulcers, chronic headaches, respiratory diseases, etc.); no psychological disorders (obsessive disorders, depression, anxiety, etc.); no previous surgery related to inflammatory bowel disease; be in latency phase, symptom-free and not taking any steroid treatment; having had the disease for 2 to 5 years.

The exclusion criteria were participants receiving other treatments, either pharmacological, psychosocial and/or complementary therapies; those with complications derived from the pathology during the research process, and/or with flare-ups and/or with complications requiring surgery; patients under 19 years old.

All participants followed daily pharmacological and dietary treatments to control the disease.

The control group complied with the following inclusion criteria: not diagnosed with inflammatory bowel disease nor any other organic or psychological disorder.

### 2.4. Ethics

The study was approved by the Ethics Commission of the Intra and External Hospital Health Research Doctorate Programme of the *Alfonso X El Sabio* University School of Medicine (Ref. 2019/19-022). A STROBE checklist was used to guide research reporting.

The samples were collected during two periods: from May 2019 to November 2019, plus a further two months to increase the sample size from November 2019 to January 2020.

### 2.5. Indicators

Two data collection instruments were used to carry out this work: the Coping Strategies Inventory by Tobin and his team [41], adapted by Cano, Rodríguez and García [42]; and the general self-efficacy scale by Schwarzer and Jerusalem [43], following the adaptation of Sanjuan, Pérez and Bermúdez [44].

#### 2.5.1. Coping Strategies Inventory by Tobin, Holroyd, Reynolds and Wigal, Adapted by Cano, Rodríguez and García

This inventory measures to what degree certain coping strategies are used, dividing them into two types. On the one hand, action-driven strategies or active coping, (i.e., engagement strategies, our active coping), that seek to compensate for the stressful situation and, on the other hand, non-action-driven strategies or passive coping (i.e., disengagement strategies, our passive coping). The Cano, Rodríguez and García adaptation [42] consists of 40 items, hierarchically organised into 8 primary, 4 secondary and 2 tertiary factors.

The primary scales are as follows: Problem Solving (cognitive and behavioural strategies to eliminate stress by altering or modifying the situation that caused the stress); Cognitive Restructuring (strategies used to modify the significance of the stressful situation); Express Emotions (strategies directed towards releasing the emotions that appear during the stressful situation); Social Support (strategies that refer to searching for emotional support to overcome the stressful situation); Problem Avoidance (strategies that include denial and avoidance of thoughts or behaviours related to the stressful event); Wishful Thinking (cognitive strategies that express the desire for a non-stressful reality); Self Criticism (strategies based on self-blame and self-criticism regarding the stressful situation or inadequate handling of the same); and Social Withdrawal (strategies to avoid and withdraw from friends, family, colleagues and other significant people associated with the emotional response to the stressful event).

The four secondary scales are the following: Problem-Focused Engagement = directed towards action centred upon the problem; Emotion-Focused Engagement = directed towards action centred upon emotion; Problem-Focused Disengagement = not directed towards action centred upon the problem; and Emotion-Focused Disengagement = not directed towards action centred upon emotions.

The tertiary scales arise from the empirical grouping of the secondary ones: adequate handling of circumstances-active or adaptive coping, and inadequate handling of circumstances-passive or maladaptive coping.

The questionnaire has two parts: the first part is a narrative where the participants must describe in detail and in writing, a stressful situation; the second part has forty Likert-type questions whose answers refer to the previously described stressful situation. Answers range from never used to always used. Coping strategies are determined by the highest resulting scores. The internal consistency of the tool, according to Cronbach’s alpha, ranges from values of 0.91 on the social support scale to 0.55 on the problem avoidance scale, and from 0.63 on the problem avoidance scale, to 0.89 on the self-criticism scale. We obtained a Cronbach’s alpha of 0.72 for the general inventory in this work, with an estimated test reliability using the two-halves technique and the Spearman–Brown formula of Rxx = 0.721.

#### 2.5.2. General Self-Efficacy Scale by Schwarzer and Jerusalem Adapted by Sanjuan, Pérez and Bermúdez

This scale measures the person’s perception and feelings regarding their own abilities and capacity to manage possible stressful situations, according to the confidence that person has in being able to deal with these, thereby obtaining a stable feeling about the matters in question and their personal ability to handle stressful situations.

The original scale is made up of 10 items that express the confidence level with which to face stressful situations. The response to these items is Likert-type, with 4 alternative responses (1 to 4). In the adaptation used for this research, the score is assessed on a 10-point scale, with a minimum score of 10 and a maximum of 100 points, with a higher score being related to greater perceived general self-efficacy. This adaptation showed high reliability thanks to its elevated internal consistency, Cronbach’s alpha coefficient of 0.87 and a correlation between two halves of 0.88. The internal consistency of the instrument in our study was α = 0.95, and the reliability of the test estimated using the two-halves technique and the Spearman–Brown formula was Rxx = 0.968.

### 2.6. Statistical Analysis

Age description was carried out via an analysis of central tendency measures (average) and dispersion (standard deviation).

Quantitative variables were analysed using a normality test (Kolmogorov–Smirnov) and using parametric tests (ANOVA, Student’s *t*-test and Pearson’s correlation coefficient). To explore the self-efficacy and coping variables studied, a comparative study was carried out between the different groups. The Levene test was used to verify the equality of variances. As regards the statistical analysis of the variables in the groups, the Tukey test was used when equality of variance between the groups existed, and the Games–Howell test in the event that this was not the case.

Finally, to discover the relationship between the two primary coping strategies (adaptive and maladaptive coping strategies), a Pearson correlation analysis was performed, and the relationship between self-efficacy with each of these two strategies (adaptive and maladaptive coping strategies), was also studied. The analysis was performed on both the patient group and the healthy participants group.

The Statistical Package for Social Sciences tool, Version 23 (SPSS 23), was used to carry out the descriptive statistics and hypothesis contrast of the study, as well as the analyses of internal consistency, reliability and validity of the instruments used in this work. In all tests, a confidence level of 95% and a *p*-value below 0.05 were considered significant.

## 3. Results

### 3.1. Description of the Sample

The sample consisted of 92 people, of whom 33 had been diagnosed with Crohn’s disease, 23 with ulcerative colitis and 36 were healthy participants (Table 1). Of these, 44.57% were men and 55.43% women. With respect to the average age of the samples, no statistically significant differences existed in people with Crohn’s disease (40.91 ± 9.15), people with ulcerative colitis (43.26 ± 9.40) and healthy people (40.28 ± 7.5).

### 3.2. Results: Self-Efficacy and Coping

#### 3.2.1. Self-Efficacy

The average self-efficacy score of healthy participants (76.72 ± 11.97), was compared to the average of participants with inflammatory bowel disease (71.38 ± 16.48), and no statistically significant differences were found between both sets of data (*p* = 0.123). Neither were statistically significant differences found when the data of the three groups were stratified as follows: patients with Crohn’s disease (69.67 ± 17.06), patients with ulcerative colitis (73.83 ± 15.65) and healthy participants (76.72 ± 11.97), (*p* = 0.149). All participants analysed were considered to be self-efficient.

#### 3.2.2. Coping: Eight Primary Factors

Upon comparison of the averages of the group of diagnosed patients and the group of healthy individuals, statistically significant differences were found for social withdrawal (*p* < 0.001) (Table 2 and Supplementary Figure S1).

When comparing the averages of the groups (Crohn’s disease, ulcerative colitis and healthy patients), statistically significant differences were found in the variables for social support (*p* = 0.030), specifically between the ulcerative colitis and healthy groups according to Tukey’s post hoc test (*p* = 0.020); for social withdrawal (*p* = 0.001), the differences were between the Crohn’s disease and healthy participants groups (*p* = 0.001), and between patients with ulcerative colitis and healthy participants (*p* = 0.030) (Supplementary Table S1).

#### 3.2.3. Coping: Four Secondary Factors

Upon comparing the group averages, statistically significant differences were found in emotion-focused engagement between the group of diagnosed patients and the group of healthy individuals (*p* = 0.044), as well as emotion-focused disengagement (*p* = 0.004) (Table 3 and Supplementary Figure S2).

Upon stratifying the groups and comparing their averages (Crohn’s disease, ulcerative colitis and healthy participants), statistically significant differences were found in the emotion-focused disengagement variable (*p* = 0.01), specifically in the averages of this strategy between the Crohn’s disease group and the healthy participants group, according to the Games–Howell post hoc test, *p* = 0.01. Supplementary Table S2.

#### 3.2.4. Coping: Two Tertiary Factors

When comparing the group averages, statistically significant differences were seen in passive coping (*p* = 0.01). The control group obtained a lower average score in passive coping than the group of diagnosed patients (Table 4 and Supplementary Figure S3).

These differences were not transferred when comparing the group averages (Crohn’s disease, ulcerative colitis and healthy patients), nor in the active or adaptive coping factor (*p* = 0.200) or in the passive or maladaptive coping factor (*p* = 0.050). However, we observed that the latter type of strategy was less utilized in healthy participants than in the group with Crohn’s disease or ulcerative colitis. Supplementary Table S3 establishes the almost significant differences (0.053).

### 3.3. Relationship between Coping Strategies

By grouping the coping strategies into two groups (adaptive strategies and maladaptive strategies), an inverse relationship between the scores of both variables corresponding to the total sample was found (r = −0.3, *p* = 0.002), indicating that participants who scored highly for active or adaptive coping, tended to have a low score for passive or maladaptive coping.

Nonetheless, upon studying this relationship separately in the three groups, it was not statistically significant for the group of healthy participants (r = −0.1, *p* = 0.400), but it was, however, statistically significant for the group of people with inflammatory bowel disease (r = −0.3, *p* = 0.007).

### 3.4. Relationship between Self-Efficacy and Coping

Taking the two tertiary factors as a reference, self-efficacy and coping strategies are related to each other in the entire sample. Specifically, self-efficacy is directly related to action-driven or adaptive coping (r = 0.3, *p* = 0.006), whilst it is inversely related to passive or maladaptive coping (r = −0.3, *p* = 0.003).

Upon studying this relationship separately for the two groups (healthy participants and patients with inflammatory bowel disease), it was also found that the relationship between self-efficacy and coping strategies is statistically significant in the case of patients with inflammatory bowel disease. In this group, the correlation of self-efficacy with active coping is direct (r = 0.3, *p* = 0.007), and with passive coping, it is inverse (r = −0.3, *p* = 0.020).

## 4. Discussion

Upon examining the results, we saw that people with inflammatory bowel disease used different coping strategies than healthy people.

As regards self-efficacy, we expected to find that healthy participants would score higher than those diagnosed with inflammatory bowel disease, as did Vinaccia and his team [27]. In our results, however, there were no statistically significant differences between healthy people and inflammatory bowel disease patients (mean of 76.72 ± 11.97 vs. 71.38 ± 16.48, *p* = 0.123). This may be because their disease was in latency phase and, therefore, the patients found themselves in very similar circumstances to those of any healthy person. Our data also indicated that both healthy participants (control group) as well as patients (case group) considered themselves to be self-efficient. The high level of self-efficacy perceived by people with inflammatory bowel disease could be due to the fact that they were in latency phase. This matter is related to learning about and handling the disease which, based on previous relapse situations, has led these patients to know the importance of controlling and managing their pathology to increase their possibilities of avoiding new flare-ups and hospital admittance. As regards latency-phase Crohn’s patients, our previous studies verified that when the external locus of control was low, this variable, together with stress, were variables that influenced the development of outbreaks [3,39]. Additionally, when patients’ efforts were directed at controlling the pathology following the recommended treatment such as diet, medication, habits and so forth the probability of experiencing a flare-up was reduced. This study not only adds to this pathology management, but also to the fact that patients feel capable of carrying out all those actions that stimulate good management of their disease.

With respect to coping, the analysis we carried out upon the primary factors reflects the existence of significant differences in the use of the social withdrawal strategy (Table 2). Generally, groups with inflammatory bowel disease used this strategy more than healthy participants. On the other hand, upon analysing the different health groups separately (Crohn’s disease, ulcerative colitis and healthy patients), we observed that patients with ulcerative colitis used the social support strategy less than the control group (Table S1). In addition, according to Tukey’s post hoc test, significant differences were also found with respect to social withdrawal, specifically between Crohn’s disease patients and healthy participants, as well as ulcerative colitis patients and healthy participants. Social support is essential for chronic pathologies since it facilitates adjustment to the illness and influences adherence to treatment and psychosocial adaptation to the illness [24,45]. Additionally, it influences behaviour with respect to treatment adherence [26]. We can therefore confirm that these people require greater use of the social support strategy and less of the social withdrawal strategy in order to experience greater adjustment, psychosocial adaptation and better management of inflammatory bowel disease.

Our results could be related to the fact that inflammatory bowel disease is a complex, chronic pathology that creates an uncomfortable and difficult situation. Therefore, people with this illness tend to avoid social contact to reduce any awkward situations [3].

The precise goal of the pre-pregnancy programme developed for women with inflammatory bowel disease by Lee and colleagues was to improve the social interaction of these women since social avoidance was also identified [46].

Regarding coping according to the four secondary factors established in the scale utilized, our results show significant differences in emotion-focused engagement coping strategies and emotion-focused disengagement (Table 3).

Our study reveals that significant differences exist between healthy participants and those diagnosed with inflammatory bowel disease, as regards the strategy directed towards action centred upon emotion (adaptative or active) (Table 3). Healthy participants used this strategy more than patients with inflammatory bowel disease. These results concur with those found by Ahadi and his group who, in their case–control study carried out upon cancer patients, found that people with cancer used the emotion-focused action-driven strategy less than healthy participants [47]. The opposite is true for the non-action-driven centred upon emotions strategy. Our results indicate that healthy people make lesser use of this coping strategy than those diagnosed with inflammatory bowel disease.

In this study, we found that strategies focused on emotion were statistically significant and were the most frequently used by people with inflammatory bowel disease.

The research carried out by Iglesias–Rey et al. in 2013 [48], who showed that participants with inflammatory bowel disease predominantly used emotion-focused coping. Additionally, research carried out by Díaz Acosta in 2015 [49] found that groups with chronic diseases and limb amputation used emotion-focused passive coping strategies more frequently. As mentioned earlier, passive coping requires less effort than active coping, so our results are possibly due to this fact.

With respect to the analysis of tertiary coping factors, statistically significant differences were found for passive coping (Table 4). Our results indicate that the control group of healthy participants used this type of coping mechanism less than the case group with inflammatory bowel disease. These results are along the same lines as those found by Grodzinsky et al. [50], who demonstrated that people with inflammatory bowel disease used less effective coping strategies than other patients with asymptomatic chronic diseases. The works of Darnopiha also showed that participants with Crohn’s disease tended to use passive coping strategies, an issue that contributes to greater psychological discomfort and worse adaptation to the pathology [35]. The use of passive coping implies poor adjustment to the disease and makes adaptation to it more complicated [45]. However, less psychological effort is required.

As regards coping strategies in general, an inverse relationship was observed between the active and passive coping strategy scores for everyone. People scoring high for the use of adaptive or active coping strategies scored low for maladaptive or passive coping strategies. This confirms that people with a high active coping score when facing stressful situations use strategies that resolve the issues. They direct their efforts at actively dealing with stressful situations and usually do so in the same way. The opposite happens with those that score high for passive coping when facing stressful situations.

Lastly, and with respect to self-efficacy and coping, our data indicated that a direct relationship existed between the self-efficacy score and the use of active coping strategies, whilst the opposite occurred as regards the use of passive coping strategies. Therefore, participants who score high in self-efficacy also score high in active or adaptive coping, whilst those who score low in self-efficacy score high in passive or maladaptive coping.

The work of Godoy–Izquierdo et al. demonstrated that high self-efficacy improves coping with stressful situations [51], and the reason for this is that people who are confident in their abilities can cope with difficult situations in a better way. Therefore, self-efficacy is related to positive coping strategies.

There are several clinical applications that can be derived from this work. First of all, actions aimed at patients’ social lives should be considered, encouraging them to expand their social circle in order to relate more, and at the same time, receive greater social support. Thus, these people would adapt better to the disease and they would have better adherence to treatment.

Secondly, actions should be put in place for individuals using emotion-focused coping strategies, to help them switch to using problem-focused strategies instead. This would allow them to benefit from the positive psychological outcomes such change implies.

Thirdly, actions should be taken to enable these patients to make more use of active coping strategies, thus helping them to direct all their efforts toward the resolution of the problems causing them stress. This would help patients adapt better to the disease and reduce their symptoms.

Finally, it is important that actions which contribute to increasing self-confidence and the patients’ ability to handle stressful situations be put in place. This would mean that they no longer see situations as threatening, thereby reducing their stress level and developing a greater capacity to adapt to their illness.

All the above are extremely important to help people with inflammatory bowel disease adapt and cope with a changing and stressful environment, thereby increasing the latency phase of their illness and reducing the frequency of flare-ups. This in turn would avoid hospital admissions and allow inflammatory bowel disease patients to lead a normal life without the frequent interruptions caused by their disease. As a whole, these actions would contribute to achieving greater success in the treatment of inflammatory bowel disease.

## 5. Conclusions

Drawn from our study is the need to strengthen social support for people with Crohn’s disease and ulcerative colitis. These people use emotion-focused strategies more frequently than problem-focused strategies. Patients with Crohn’s disease and ulcerative colitis generally use more passive coping than active coping. We see an inverse relationship between the use of active coping and passive coping in these patients. Finally, the level of self-efficacy correlates positively with the use of active coping strategies and vice versa.

## 6. Strengths and Weaknesses

The greatest strength of our study is having shown that people with inflammatory bowel disease make greater use of passive coping strategies than healthy people. In addition, that self-efficacy levels are important for the use active coping strategies.

Another strength is having demonstrated that people with inflammatory bowel disease make greater use of emotion-focused strategies than healthy participants. Additionally, it is important that they develop their social circles to effectively leave behind the social withdrawal coping strategy and make greater use of the social support coping strategy.

As regards the limitations or weaknesses of our study, we found the greatest one to be our sample size. For this reason, we suggest that our conclusions be considered carefully, and we feel it is necessary to confirm these results in future research using larger samples.

## Figures and Tables

**Table 1:** Distribution of the sample.

Diagnosis	Average	Gender		Total *n* (%)
		Male *n* (%)	Female *n* (%)	
Crohn’s disease	40.91	18 (19.6%)	15 (16.3%)	33 (35.9%)
Ulcerative colitis	43.26	7 (7.6%)	16 (17.4%)	23 (25.0%)
Healthy participants	40.28	26 (28.3%)	10 (10.9%)	36 (39.1%)

**Table 2:** Independent sample *t*-test results of the total score in the eight primary coping factors according to diagnosis [people with inflammatory bowel disease (*n* = 56) vs. healthy participants (*n* = 36)].

Coping Mechanism	Diagnosis	M	SD	*p*-Value
Problem Solving	Diagnosed	15.80	4.08	0.842
	Healthy	15.97	3.72	
Cognitive Restructuring	Diagnosed	11.71	4.80	0.508
	Healthy	12.39	4.66	
Emotional Expression	Diagnosed	9.79	4.62	0.102
	Healthy	11.36	4.21	
Social Support	Diagnosed	11.98	4.63	0.075
	Healthy	13.67	3.94	
Problem Avoidance	Diagnosed	6.73	4.07	0.392
	Healthy	7.47	3.94	
Wishful Thinking	Diagnosed	14.05	5.27	0.161
	Healthy	12.47	5.17	
Self-Criticism	Diagnosed	7.30	6.58	0.126
	Healthy	5.33	4.87	
Social Withdrawal	Diagnosed	8.30	5.07	<0.001
	Healthy	4.47	4.17	

Values expressed as mean (M) and standard deviation (SD), *p* value of less than 0.05 were considered significant.

**Table 3:** Independent sample *t*-test results of the total score in the four secondary coping factors according to diagnosis [people with inflammatory bowel disease (*n* = 56) vs. healthy participants (*n* = 36)].

Coping Mechanism	Diagnosis	M	SD	*p*-Value
Problem-focused Engagement	Diagnosed	27.52	7.28	0.586
	Healthy	28.36	7.12	
Emotion-focused Engagement	Diagnosed	21.77	7.75	0.044
	Healthy	25.03	7.00	
Problem-focused Disengagement	Diagnosed	20.79	5.81	0.489
	Healthy	19.94	5.45	
Emotion-focused Disengagement	Diagnosed	15.61	10.14	0.004
	Healthy	9.81	7.74	

Values expressed as mean (M) and standard deviation (SD), *p* value of less than 0.05 were considered significant.

**Table 4:** Independent sample *t*-test results of the total score in both tertiary coping factors according to diagnosis [people with inflammatory bowel disease (*n* = 56) vs. healthy participants (*n* = 36)].

Coping Mechanism	Diagnosis	M	SD	*p*-Value
Active Coping	Diagnosed	49.29	12.43	0.113
	Healthy	53.39	11.31	
Passive Coping	Diagnosed	36.39	13.92	0.017
	Healthy	29.77	10.70	

Values expressed as mean (M) and standard deviation (SD), *p* value of less than 0.05 were considered significant.

## Data Availability

Not applicable.

## Supplementary Materials

The following supporting information can be downloaded at: https://www.mdpi.com/article/10.3390/healthcare11081113/s1, Table S1: Total score of each of the eight primary factors per diagnosis (Crohn’s disease vs. Ulcerative colitis vs. Healthy); Table S2: Total score of the four secondary factors per diagnosis (Crohn’s disease vs. Ulcerative colitis vs. Healthy); Table S3: Total score of both tertiary factors per diagnosis (Crohn’s disease vs. Ulcerative colitis vs. Healthy). Figure S1: Total score of each of the eight primary coping factors according to diagnosis [people with inflammatory bowel disease (*n* = 56) vs. healthy participants (*n* = 36)]; Figure S2: Total score of each of the four secondary coping factors according to diagnosis [people with inflammatory bowel disease (*n* = 56) vs. healthy participants (*n* = 36)]; Figure S3: Total score of both tertiary coping factors according to diagnosis [people with inflammatory bowel disease (*n* = 56) vs. healthy participants (*n* = 36)].
