# Supplementary material for: Influence of Coping and Self-Efficacy in Inflammatory Bowel Disease
Source: Healthcare (Basel). 2023 Apr 13;11(8):1113. doi: 10.3390/healthcare11081113 (PMC10138294; doi:10.3390/healthcare11081113)
Supplement: Supplementary file 1 [file healthcare-11-01113-s001.zip › healthcare-2224683-supplementary.pdf]

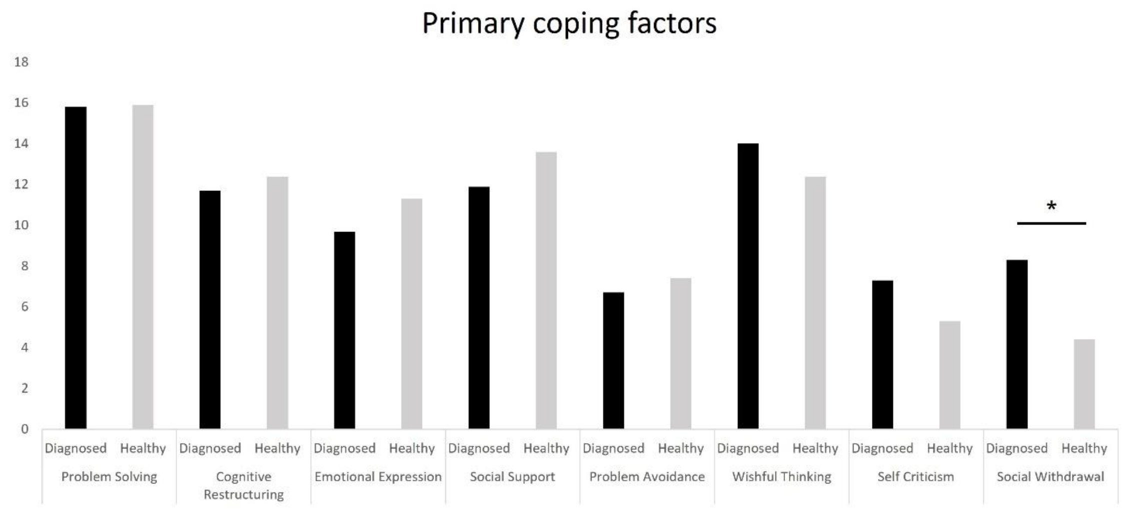

* $p < 0.05$

Figure S1: Total score of each of the eight primary coping factors according to diagnosis [People with inflammatory bowel disease (n = 56) vs Healthy subjects (n = 36)]

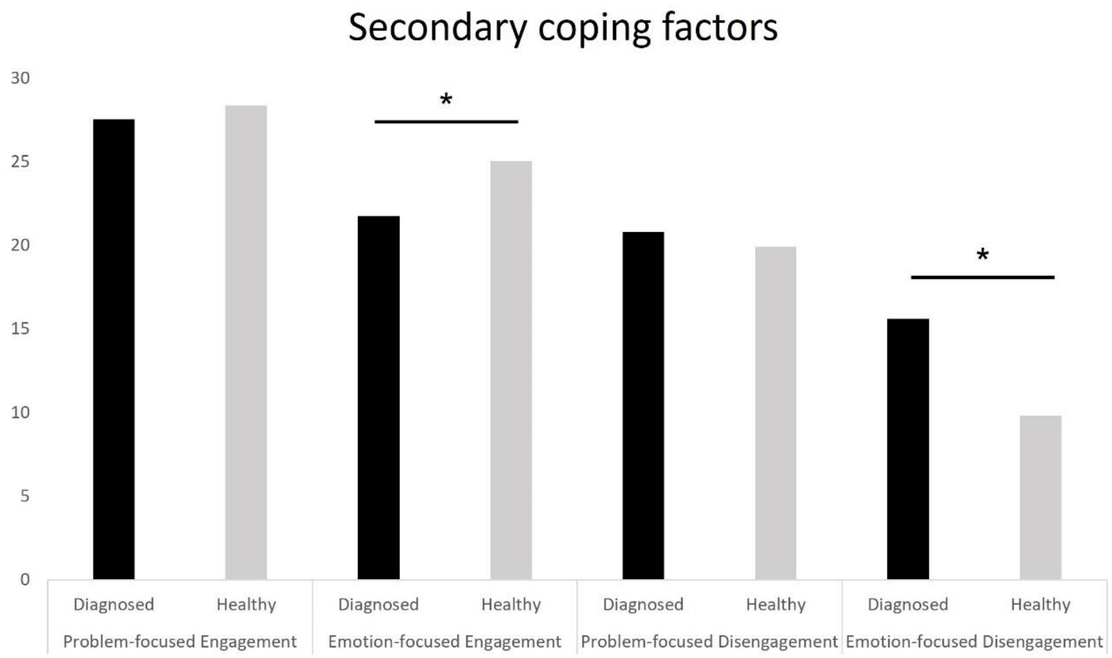

* $p < 0.05$

Figure S2: Total score of each of the four secondary coping factors according to diagnosis [People with inflammatory bowel disease (n = 56) vs Healthy subjects (n = 36)]

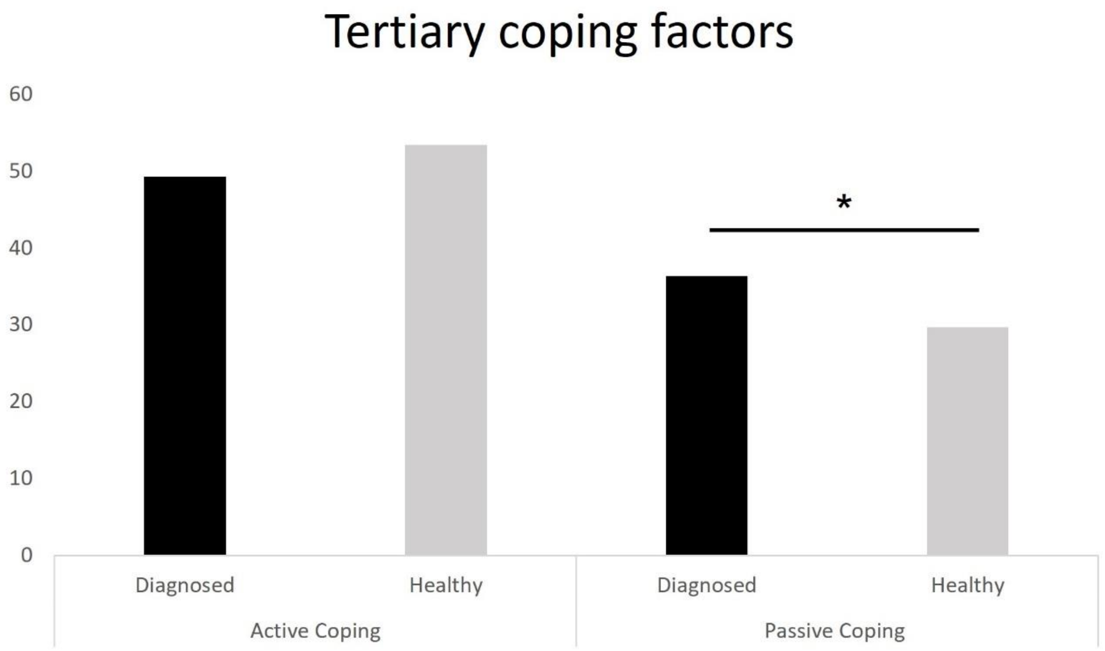

* $p < 0.05$

Figure S3: Total score of both tertiary coping factors according to diagnosis [People with inflammatory bowel disease (n = 56) vs Healthy subjects (n = 36)]
