# Supplementary material for: Influence of Coping and Self-Efficacy in Inflammatory Bowel Disease
Source: Healthcare (Basel). 2023 Apr 13;11(8):1113. doi: 10.3390/healthcare11081113 (PMC10138294; doi:10.3390/healthcare11081113)
Supplement: Supplementary file 1 [file healthcare-11-01113-s001.zip › Suplementary Materials-.pdf]

Table S1. Total score of each of the eight primary factors per diagnosis (Crohn’s Disease vs Ulcerative Colitis vs Healthy).

Coping Mechanism	Diagnosis	*M*	*SD*	*Min.*	*Max.*	*p-value*
Problem Solving	Crohn’s Disease	16.06	4.38	2.0	20.0	0.828
	Ulcerative Colitis	15.43	3.67	7.0	20.0	
	Healthy	15.97	3.72	6.0	20.0	
Cognitive Restructuring	Crohn’s Disease	11.70	4.97	0.0	20.0	0.803
	Ulcerative Colitis	11.74	4.66	4.0	20.0	
	Healthy	12.39	4.66	4.0	20.0	
Emotional Expression	Crohn’s Disease	9.67	4.56	0.0	20.0	0.257
	Ulcerative Colitis	9.96	4.80	1.0	19.0	
	Healthy	11.36	4.21	3.0	19.0	
Social Support	Crohn’s Disease	12.94	4.45	5.0	20.0	0.030 *
	Ulcerative Colitis	**10.61**	4.64	0.0	19.0	
	Healthy	**13.67**	3.94	3.0	20.0	
Problem Avoidance	Crohn’s Disease	6.36	4.10	0.0	16.0	0.149
	Ulcerative Colitis	7.26	4.06	1.0	17.0	
	Healthy	7.47	3.97	0.0	18.0	
Wishful Thinking	Crohn’s Disease	14.94	4.65	3.0	20.0	0.149
	Ulcerative Colitis	12.78	5.93	2.0	20.0	
	Healthy	12.47	5.17	1.0	20.0	
Self-criticism	Crohn’s Disease	7.03	6.43	0.0	20.0	0.149
	Ulcerative Colitis	7.70	6.92	0.0	20.0	
	Healthy	5.33	4.87	0.0	19.0	
Social Withdrawal	Crohn’s Disease	**8.67**	4.85	0.0	20.0	0.001 **
	Ulcerative Colitis	**7.78**	5.44	0.0	20.0	
	Healthy	**4.47**	4.17	0.0	18.0	

Note. Crohn’s Disease, n = 33; Ulcerative Colitis, n = 23; Healthy Subjects, n = 36; total, N = 92. ANOVA test: * $p < 0.05$ / ** $p < 0.01$ / *** $p < 0.001$

Table S2. Total score of the four secondary factors per diagnosis (Crohn's Disease vs Ulcerative Colitis vs Healthy).

Coping Mechanism	Diagnosis	*M*	*SD*	*Min.*	*Max.*	*p-value*
Problem-Focused Engagement	Crohn's Disease	27.76	7.65	8.0	40.0	0.826
	Ulcerative Colitis	27.17	6.87	13.0	40.0	
	Healthy	28.36	7.12	13.0	40.0	
Emotion-Focused Engagement	Crohn's Disease	22.61	7.96	6.0	40.0	0.080
	Ulcerative Colitis	20.57	7.43	7.0	33.0	
	Healthy	25.03	7.00	9.0	36.0	
Problem-Focused Disengagement	Crohn's Disease	21.30	5.50	11.0	33.0	0.566
	Ulcerative Colitis	20.94	6.26	7.0	30.0	
	Healthy	19.94	5.45	10.0	31.0	
Emotion-Focused Disengagement	Crohn's Disease	**15.70**	9.32	0.0	35.0	0.017 *
	Ulcerative Colitis	15.48	11.43	0.0	40.0	
	Healthy	**9.81**	7.74	0.0	31.0	

Note. Crohn's Disease, n = 33; Ulcerative Colitis, n = 23; Healthy Subjects, n = 36; total, N = 92. ANOVA test: * p <0.05 / ** p <0.01 / *** p <0.001

Table S3. Total score of both tertiary factors as per diagnosis (Crohn's Disease vs Ulcerative Colitis vs Healthy).

Coping Mechanism	Diagnosis	*M*	*SD*	*Min.*	*Max.*	*p-value*
Active Coping	Crohn's Disease	50.36	13.36	24.0	77.0	0.208
	Ulcerative Colitis	47.74	11.08	28.0	65.0	
	Healthy	53.39	11.31	27.0	74.0	
Passive Coping	Crohn's Disease	37.00	12.69	16.0	63.0	0.053
	Ulcerative Colitis	35.52	15.77	9.0	59.0	
	Healthy	29.75	10.70	13.0	52.0	

Note. Crohn's Disease, n = 33; Ulcerative Colitis, n = 23; Healthy Subjects, n = 36; total, N = 92. ANOVA test: * p <0.05 / ** p <0.01 / *** p <0.001
